# Factors influencing development of professional values among nursing students

**DOI:** 10.12669/pjms.324.10616

**Published:** 2016

**Authors:** Renginar Ozturk Donmez, Suheyla Ozsoy

**Affiliations:** 1 Renginar Ozturk Donmez, PhD, RN. Department of Public Health Nursing, Nursing Faculty of Ege University, Izmir, Turkey; 2 Suheyla Ozsoy, Professor, Department of Public Health Nursing, Nursing Faculty of Ege University, Izmir, Turkey

**Keywords:** Nursing, Student, Professional Value, Education

## Abstract

**Objective:**

To determine the professional values of Turkish nursing students, and to explore the relationships between their characteristics.

**Methods:**

The cross-sectional study participants consisted of 416 nursing students who were studying in a nursing faculty in Western Turkey. A questionnaire was used to identify socio-demographic and educational characteristics and the Nursing Professional Values Scale- Revised (NPVS-R) was used for this study..

**Results:**

The total mean score of the NPVS-R was found to be 99.45±1.96, and items mean score was 3.82±0.62. The NPVS-R score was significantly higher in students who were female, and who chose their profession willingly, had information about values, and who were members of a professional organization.

**Conclusion:**

The students were found to have strong professional values, and professional values affected some of the personal and educational characteristics of nursing students.

## INTRODUCTION

The nursing is a profession that provides 24-hour service to all persons. While providing this services, nurses encounter numerous situations that require them to make decisions.^1^,^2^ Each decision about healthy individuals or patients should be made with respect for their life, honor and individuality. A well-developed value system helps nurses find solutions to ethical dilemmas in the decision making process.^3^ As professionals providing health care services, nurses should make their decisions according to professional values.^3^,^4^ Professional values included in the classification of values are a thought system which is accepted by occupational groups and establishes a standard structure for attitudes and beliefs affecting behaviors.^5^,^6^ Developing professional values in nursing is important because of care quality and increase of patient’s understanding; it also helps the professional socialization process.^6^ Professional values take their forms together with the culture of the current setting of the nursing students. Nursing education is a long and formal education in which students are expected gain values and attitudes appropriate to the role of nursing as well as professional knowledge and clinical skill.^7^,^8^ From the first year of study to the final year, academic instructors encourage students consciously and continuously towards certain attitudes while attempting to oppose some of their attitudes.

As nursing students are future nurses, it is crucial to understand their values in managing care and component practice as well as their awareness about upholding the image of the profession upon graduation.^7^ In the literature, it was stated that determining the attitudes of nursing students towards professional values is necessary to shape present and future education strategies.^9^-^11^ In recent years, following the development of the nursing professional value scale by Weis and Schank (2000),^3^ the tendency towards examining professional values has increased worldwide.^7^,^8^,^10^-^15^ However, the professional values of nursing students has not been examined in Turkey. The aim of this study was to determine factors influencing development of the professional nursing values of nursing students in Turkey.

## METHODS

This cross-sectional study was conducted during January and March 2016 in a Nursing Faculty which was the first Bachelor of Science (B.Sc.) nursing program established in Turkey. The study population consisted of 1432 students, including 296 first -year students, 482 second-year students, 397 third-year students and 257 fourth year students, who were studying in the nursing faculty. As per Saunders et al. study^16^ the level of 5% margin of error, it is adequate to include 278 individuals and 322 individuals in populations consisting of 1000 and 2000 individuals, respectively in the sample. The total of the number of students selected from each class using stratified sampling method was determined to be at least 286 students for determining the sample size. The study sample consisted of 416 students, including 92 (22.1%) first-year students, 132 (31.7%) second-year students, 109 (26.2%) third-year students, and 83 (20.0%) final- year students, who were studying in the nursing faculty. The inclusion criteria was being a student in the nursing faculty and volunteering to participate in the study.

The study data were collected using a personal information which consisted of 15 questions to specify the students’ socio-demographic and educational characteristics, and the Nursing Professional Value Scale-R (NPVS-R). The NPVS-R was developed by Weis and Schank (2009).^2^ It is a 5-point Likert-type scale consisting of 26 items, scoring of which ranges from one to five (1 = Not at all important, 5 = Most Important). The lowest possible score obtainable from the scale is 26 and the highest possible score is 130. The fact that an individual obtains a high score for the scale indicates that this individual has strong professional values. NPVS-R the validity and reliability study of which was conducted by Ozsoy and Dönmez (2015)^17^ for Turkish society. In their study, the Cronbach’s alpha coefficient of the NPVS-R was found to be 0.92, and obtained four sub-dimensions. These sub-dimensions are Professionalism, Caring, Activism, and Trust.^17^

The SPSS 16.0 program was used for the data analysis. Continuous variables were calculated as means, standard deviations, frequencies and percentages. Kolmogorov-Smirnov analysis was used to determine the suitability of data to the normal distribution. Comparisons were made using the Mann Whitney U tests and the Kruskal Wallis tests. The level of significance in all analyses was set at p < 0.05. It was approved by the Ethics Committee of the Ege University Nursing Faculty and a verbal consent was received from the students.

## RESULTS

A total of 416 B.Sc. nursing students completed questionnaires. Of them, 81.0% were female and 19.0% were male. Their average age was 20.93±1.65, and their age groups were 54.1% between 21 and 23, 38.5% between 18 and 20, 6.7% over 24. Of them, 44.2% had lived in city, 34.1% had lived in metropolitan, and 21.6% had lived in village for a long time.

Of the students, 57.9% decided to study nursing by their own will, while 42.1% were not willing to study nursing. They were found to prefer to study nursing for reasons such as having a guarantee of a job (47.6%), loving the nursing profession (27.4%), their family’s desire (16.6%), and the low scores obtained from university entrance exam (8.4%). Over half of the students, 50.5%, were planning to work as clinician nurses after their graduation while 49.5% were planning to work as academics. The researchers posed yes–no questions to students about their preliminary information about the values, membership to professional associations and social organizations. Thus, 50.7% of them had received information about values before, and almost all students received this information from the courses provided by the faculty. Of the nursing students, 32.2% were found to be members of professional associations, and 24.3% to be members of a non-governmental organization.

The NPVS-R total mean score of the nursing students was found to be 99.45±1.96 (26.0-130.0), and their items mean score to be 3.82±0.62. The caring sub-dimension mean score was found to be 3.94±0.69; the professionalism sub-dimension mean score to be 3.84±0.64; the trust sub-dimension mean score to be 3.78±0.69; and the activist sub-dimension mean score to be 3.61±0.66. The students were found to obtain the highest scores from the caring sub-dimension and the lowest scores from the activist sub-dimension (Table-I).

**Table-I:** Homogeneity, mean and rankings of NPVS-R.

	Kolmogorov-Smirnov	p	Mean±SD	Ranking
The NPVS-R total mean score	0.72	0.00	99.45±1.96	-
The NPVS-R items mean score	0.72	0.00	3.82±0.62	-
Sub-dimensions				
Professionalism	0.78	0.00	3.84±0.64	2
Caring	0.12	0.00	3.94±0.69	1
Activist	0.10	0.00	3.61±0.66	4
Trust	0.14	0.00	3.78±0.69	3

SD: Standart deviation

The female students were found to obtain higher scores from the NPVS-R total score compared to the male students, and also to have stronger professional values (p < 0.05). The students aged between 18 and 20 were found to obtain the highest scores from the NPVS-R while the students aged 24 and above were found to obtain the lowest score from the scale’s total score (p < 0.05). The students were found to have weaker professional values as their age increased (Table-II).

**Table-II:** Comparison of the total NPVS-R items means scores by various characteristics

Characteristics	The total of NPVS-R items mean		

	Mean ±SD	Test	p
***Academic year***			
First year	3.84±0,60	x^2^=1.054	p=0.078
Second year	3.83±0.61		
Third year	3.82±0.63		
Fourth year	3.77±0.62		
***Gender***			
Female	3.86 ±0.59	U=109.000	p=0.017
Male	3.66±0.68		
***Age groups***			
18-20 ages	3.87±0.54	x^2^=14.620	p=0.002
21-23 ages	3.84±0.64		
≤24 ages	3.44±0.69		
***Reason for entering nursing school***			
Love the nursing profession	3.90±0.55	x^2^=3.809	p=0.432
Low scores at the university entrance exam	3.80±0.53		
Families’ desire	3.79±0.71		
Guarantee of finding a job	3.78±0.63		
***Working field after graduation***			
Clinician nurse	3.74±0.58	x^2^=2.582	p=0.275
Academician nurse	3.85±0.68		
Other	3.89±0.53		
***Intentional choice of nursing***			
Yes	3.88±0.60	U=176.000	p=0.012
No	3.74±0.63		
***Membership of professional associations***			
Yes	3.90±0.65	U=162.000	p=0.036
No	3.78±0.59		
***Receiving information about values***			
Yes	3.91±0.60	U=178.000	p=0.005
No	3.73±0.62		

SD: Standart deviation, x^2^: Kruskall Wallis test, U: Mann Whitney U test

The NPVS-R mean score of the students who intentionally chose the nursing profession were found to be higher than those of the students who did not (p < 0.05). The students who intentionally chose the nursing profession were found to have stronger professional values. The NPVS-R mean score of the students who were members of professional associations were found higher than those of the students who were not (p < 0.05). The students who were members of professional associations were found to have stronger professional values. The NPVS-R mean score of the students who having information about values already were found to be higher than those of the students who does not (p < 0.05) (Table-II).

The students’ academic year, their reason for preferring the nursing profession, and the field they wanted to work in after their graduation were found not to make a statistically significant difference in the NPVS-R mean score (p > 0.05) (Table-II).

## DISCUSSION

Understanding baccalaureate nursing students’ attitudes about professional nursing values, including their perceptions of the importance and difficulty of application regarding all aspects of professional conduct, may provide valuable information for nurse educators in designing more effective teaching strategies.^10^ The professional values of the nursing students, who were studying at the first B.Sc. nursing faculty established in Turkey, and the factors affecting their professional values were determined in the present study. Since professional education, culture, and socio-demographic characteristics play an important role in shaping the professional values of nursing students the findings of the present study are thought to be important.^8^,^13^,^14^

Studies conducted in different countries have also found nursing students to have high levels of professional values, as in the present study.^9^,^11^,^14^ Schank and Weis (2001)^4^ stated that the fact that nursing students and nurses who had recently started work were not cognizant of professional values and behaviors was frequently pointed out. That Lin et al. (2010)^18^ stated that the nurses NPVS-R scored 100.01±15.61 indicates that the importance attached by nursing students to professional values was not very different from the importance attached by nurses.

In the present study, the nursing students were found to obtain the highest score from the caring sub-dimension and the lowest score from the activist sub-dimension. Parvan et al. (2012)^12^ in Iran, Alfred et al. (2011)^13^ in Taiwan, Lin et al. (2010)^18^ in China, and Leners et al. (2006)^19^ in United States have reported that the most important sub-dimension for nursing students is the caring sub-dimension, as in the present study. Alfred et al. (2011)^13^ found that the most important sub-dimension for nursing students in USA was the activist subdimension, while Bang et al. (2013)^14^ found that the most important sub-dimension for nursing students in Korea was the professionalism sub-dimension. Parandeh et al. (2014)^11^ stated that culture plays an important role in giving shape to values. In terms of perceiving the activist sub-dimension, this result was also regarded as a reflection of the sociopolitical process experienced by the Middle East on the students. However, most of the studies found that the most important statements for nursing student were in the caring sub-dimension since caring for patients in a professional manner is main focus of the code of ethics for nurses.^12^,^13^,^14^,^15^,^20^ Patient care is a mandatory aspect of nursing education and students understand the caring factor to be the most important value. 7

The total scale scores of the female students were found to be higher compared to those of the male students, and the female nursing students were found to have stronger professional values in our study. Other studies have reported results similar to the present study, that female nursing students have stronger professional values compared to male nursing students in Iran, Korea and USA.^12^,^14^,^20^,^21^ However, the NPVS-R scores of male nursing students in Hong Kong and Taiwan were found higher than those of female students.^10^,^13^ In Turkey, following the amendment introduced to the Nursing Law in 2007,^22^ males became entitled to work as nurses. It was evaluated that male nurses in Turkey have lower levels of professional values because they have only recently entered into the profession and are not yet used to the profession which has long been attributed as a female occupation. However, it will be necessary for the male students’ awareness about professional values to be increased because the number of male nurses and nursing students will increase in faculties and working areas.

In the present study, the students aged 24 and above were found to obtain lower scores compared to those in other (younger) age groups in terms of the scale total score. There are references in literature showing that as age increases, professional values either increase, decrease, or do not change.^20^,^21^ In accordance with these references, it was found that the effect of the nursing students’ age on their attitudes towards professional values was not determinative.

The NPVS-R score of the students who intentionally chose the nursing profession were found to be higher and they were found to have stronger professional values. It can be stated that nursing students in general tend towards nursing with similar motivation. Although the most important factor in the choice of nursing profession is that it provides a guarantee of finding a job, it is significant that the statements which were regarded as important by the students were under the title of the caring subdimension. Even though the students preferred the nursing profession because it provides a guarantee of finding a job, it can be concluded that they basically tended to join this profession with the intention of loving and helping individuals.

Students who were members of any professional associations embraced more strongly the values. It is an expected and consistent result, since the items in the scale include defending the patient and participating in the activities of professional associations. Studies about values did not examine the relationship of being a member of any professional associations and the professional values of nursing students.

It is stated in the literature that additional information about professional education and values has a positive effect on the development of values.^5^,^11^,^14^,^15^ In parallel with the literature, it was found in the present study that already having information about values was a variable, increasing the NPVS-R total score.

In the present study, the students’ year of academic, reason for preferring nursing profession, and the field they wanted to work after their graduation were found not to affect the importance level of their nursing profession values. Similarly, studies conducted on different societies found that the students’ year of academic and reason for preferring the nursing profession did not have a significant effect on their professional values.^12^,^14^,^21^ It can be concluded that nursing students were inclined to join this profession with their intrinsic motivation.

## CONCLUSION

Developing professional values among nursing students is important since such values are a significant predictor of quality care and development of profession. Study showed that the nursing students had strong professional values. The students obtained the highest score from the caring subdimension and the lowest score from the activist subdimension. The students, who were female, aged between 18 and 20, who had intentionally chosen the profession, were a member of a professional association, and had information about values already, were found to have stronger professional values. The reasons why nursing students give less priority to the trust, activist, and professionalism subdimensions than the caring subdimension could be examined in qualitative studies.

## References

[1] Uslusoy EC, Gurdogan EP, Aydinli A (2015). Professional values of Turkish nurses: a descriptive study. Nurs Ethics.

[2] Weis D, Schank JM (2009). Development and psychometric evaluation of the nurses professional values scale-revised. J Nurs Meas.

[3] Weis D, Schank JM (2000). An instrument to measure professional values. J Nurs Sch.

[4] Shank JM, Weis D (2001). Service and education share responsibility for nurses’ value development. J Nurses Staff Dev.

[5] Fry ST, Johnstone MJ (2008). Ethics in nursing practice. A guide to ethical decision making.

[6] Horton K, Tschudin V, Forget A (2007). The value of nursing: A literature review. Nurs Ethics.

[7] Jimenez-Lopez FR (2014). Values in nursing students and professionals: An exploratory comparative study. Nurs Ethics.

[8] Thorpe K, Loo R (2003). The values profile of nursing undergraduate students: Implications for education and professional development. J Nurs Educ.

[9] Iacobucci TA, Daly BJ, Lindell D, Griffin MQ (2012). Professional values, self-esteem, and ethical confidence of baccalaureate nursing students. Nurs Ethics.

[10] Lui MHL, Lam LW, Lee IFK, Chien WT, Chau JPC, Ip WY (2008). Professional nursing values among baccalaureate nursing students in Hong Kong. Nurse Education Today.

[11] Parandeh A, Khaghanizade M, Mohammadi E, Mokhtari-Nouri J (2014). Factors influencing development of professional values among nursing students and instructors: a systematic review. Glob J Health Sci.

[12] Parvan K, Zamanzadeh V, Hosseini FA (2012). Assessment of professional values among Iranian nursing students graduating in universities with different norms of educational services. Thrita.

[13] Alfred D (2011). A Comparison of professional values of Taiwanese and American nursing students. https://stti.confex.com/stti/bc41/webprogram/Paper48606.html.

[14] Bang KS, Kang JM, Jun JM, Kim HS, Son HM, Yu SJ, Kim JS (2011). Professional values in Korean undergraduate nursing students. Nurse Educ Today.

[15] Fisher MD (2014). A Comparison of professional value development among pre-licensure nursing students in associate degree, diploma, and Bachelor of Science in nursing programs. Nurs Educ Perspect.

[16] Saunders M, Lewis P, Thornhill A (2009). Research methods for business students. Selecting samples. Pearson Education, England.

[17] Ozsoy S, Dönmez RO (2015). Nurses professional values scale-revised: Psychometric properties of the Turkish version. Nurs Pract Today.

[18] Lin YM, Wang L (2010). A Chinese version of the revised nurses professional values scale: Reliability and validity assessment. Nurse Educ Today.

[19] Leners DW, Roehrs C, Piccone AV (2006). Tracking the development of professional values in undergraduate nursing students. J Nurs Educ.

[20] Martin P, Yarbrough S, Alfred D (2003). Professional values held by baccalaureate and associate degree nursing students. J Nurs Sch.

[21] Hayes TL (2007). An exploration of professional values held by baccalaureate and associate degree nursing students.

[22] Official Journal (2007). Law regarding nursing law changes. Number: 5634.

